# Promoting Photocatalytic Activity of NH_2_-MIL-125(Ti) for H_2_ Evolution Reaction through Creation of Ti^III^- and Co^I^-Based Proton Reduction Sites

**DOI:** 10.1021/acsami.3c15490

**Published:** 2023-11-15

**Authors:** Vitalii Kavun, Evgeny Uslamin, Bart van der Linden, Stefano Canossa, Andrey Goryachev, Emma E. Bos, Jara Garcia Santaclara, Grigory Smolentsev, Eveliina Repo, Monique A. van der Veen

**Affiliations:** † Department of Separation Science, LUT University, FI-53850 Lappeenranta, Finland; ‡ Department of Chemical Engineering, Delft University of Technology, 2629 HZ Delft, The Netherlands; § Department of Nanochemistry, Max Planck Institute for Solid State Research, 70569 Stuttgart, Germany; ∥ Paul-Scherrer Institute, CH-5232 Villigen PSI, Switzerland

**Keywords:** hydrogen evolution, visible light, metal–organic frameworks, photocatalysis, cobalt

## Abstract

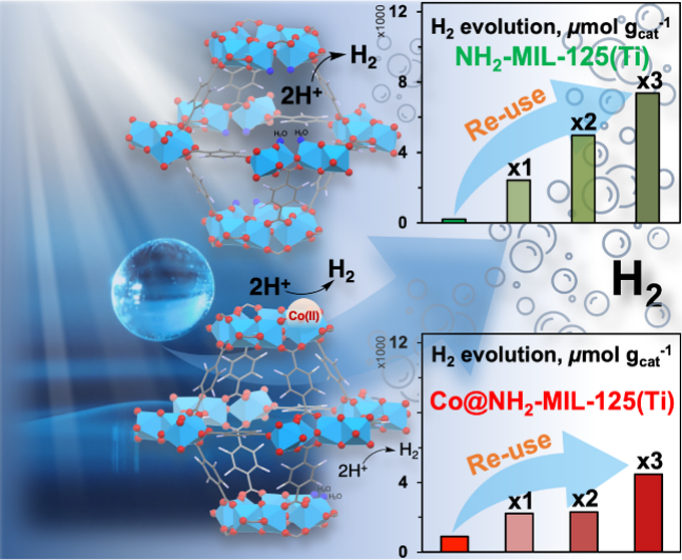

Titanium-based metal–organic framework, NH_2_-MIL-125(Ti), has been widely investigated for photocatalytic applications but has low activity in the hydrogen evolution reaction (HER). In this work, we show a one-step low-cost postmodification of NH_2_-MIL-125(Ti) via impregnation of Co(NO_3_)_2_. The resulting Co@NH_2_-MIL-125(Ti) with embedded single-site Co^II^ species, confirmed by XPS and XAS measurements, shows enhanced activity under visible light exposure. The increased H_2_ production is likely triggered by the presence of active Co^I^ transient sites detected upon collection of pump-flow-probe XANES spectra. Furthermore, both photocatalysts demonstrated a drastic increase in HER performance after consecutive reuse while maintaining their structural integrity and consistent H_2_ production. Via thorough characterization, we revealed two mechanisms for the formation of highly active proton reduction sites: nondestructive linker elimination resulting in coordinatively unsaturated Ti sites and restructuring of single Co^II^ sites. Overall, this straightforward manner of confinement of Co^II^ cocatalysts within NH_2_-MIL-125(Ti) offers a highly stable visible-light-responsive photocatalyst.

## 1 Introduction

Increasing concerns about climate change and reliance on fossil fuel sources push forward the goal of a ubiquitous deployment of renewable energy technologies and accelerate production and transition to clean carbon-free fuels.^1^ Converting solar energy into a chemical fuel, for example, by performing the photocatalytic water splitting reaction to produce molecular hydrogen, appears to be one of the most promising and green ways. However, to efficiently drive the hydrogen evolution reaction (HER), the development of light-responsive, durable, and cost-efficient photocatalysts is necessary.

Metal–organic frameworks (MOFs), which consist of metal ions and charged linkers self-assembled into a 3-D crystalline nanoporous material, have attracted significant attention in many applications. The large variation of possible building blocks implies high tunability of this material class. Combined with their intrinsic porosity, they have emerged as attractive alternatives for existing inorganic photocatalysts.^2^ To date, many frameworks based on different metals, such as Fe, Ti, Zn, and Zr, demonstrated light-harvesting properties and were able to perform HER,^3^ CO_2_ reduction,^4^ and other photochemical reactions,^5^ yet their efficiency remains modest in comparison to existing inorganic semiconductors.^6^

The potential of MOFs for the H_2_ evolution reaction can be enhanced by a rational design and the variation of building units, which can result in more efficient light absorption and improved spatial separation of photogenerated electron–hole pairs.^2^ Different approaches, including functionalization or extension of conjugated systems of organic linkers of MOFs,^5,7^ synthesis of hybrid covalent/metal–organic frameworks,^8^ encapsulating molecular catalysts or metal nanoparticles,^9,10^ and introducing missing linker/metal cluster defects in an ideal crystalline structure,^11^ have been proposed to enhance their overall performance for the water splitting reaction. For example, a slight alteration in the content of amino functional groups in the aromatic BDC building block of MIL-125(Ti) leads to a notable shift in the MOF’s light response to the visible region.^12^ Alternatively, creating missing linker vacancies in the MOF structures, such as defect-rich Ti–O sheets of COK-47, Zr^IV^-based UiO-66, and NH_2_-MIL-125(Ti), provides an effective way to create additional reactive open metal sites to facilitate a variety of light-driven chemical reactions.^11,13−16^

A highly porous Ti-based MOF, NH_2_-MIL-125(Ti), is particularly interesting for photocatalysis due to its effective linker-to-metal charge transfer (LMCT) upon visible light excitation.^17^ Notably, the lowest unoccupied and highest occupied crystalline orbitals (LUCO-HOCO) of the MOF are localized on the inorganic cluster and organic linker, respectively. Thus, light-induced charged species can be spatially separated at the crystalline orbitals, inhibiting fast electron–hole recombination, which, in turn, should facilitate photocatalytic reactions.^18^ However, the lack of proton reduction sites on the Ti–oxo cluster leads to hampered electron transfer and relatively poor performance of NH_2_-MIL-125(Ti) in HER.^15^

Considering this, the pore space of NH_2_-MIL-125(Ti) can be utilized for the confined growth of metal nanoclusters or the inclusion of molecular metal complexes. A significant number of studies have focused on doping expensive noble nanoparticles as a cocatalyst into the pore space of the MOF.^3,19−22^ The presence of the cocatalyst significantly enhances the H_2_ evolution rate of the MOF via effective trapping of photoinduced electrons and prolonging the charge-separated state. Introducing systems with cheaper and earth-abundant *d* elements, such as Co-, Ni-, or Cu-based cocatalyst nanoparticles, can provide an alternative way of developing new types of promising photocatalysts.^23−26^ However, the incorporation of metal nanoparticles into photocatalytic structures can be accompanied by certain challenges, particularly concerning their size control and stabilization. Throughout the synthesis, they are prone to agglomerate, which may reduce their catalytic efficiency, or during the reaction, rapid deactivation of active sites by adsorption may occur. Furthermore, the presence of metal cocatalysts can result in a decrease in available surface area and pore size, potentially hindering the transport of reactants within the structure. Another aspect to consider is that the core metal atoms inside nanoparticles generally stay inactive for catalytic surface reactions, implying that the atomic efficiency of these systems is comparatively lower than that of molecular metal complexes.^27−30^

Recent developments of coordination metal complexes led to an emerging potential of single-site molecular catalysts particularly with earth-abundant metal centers, including Mn, Fe, Cu, and Ni,^31−35^ while cobalt-based complexes have become one of the most prevalent catalysts for hydrogen production, thoroughly studied both theoretically and experimentally.^32,36^ Despite their promising photoactivity, the wider application of cobalt-based complexes was slowed down by their structural fragility, limited water solubility, narrow operational pH range, and inclination to form nanoparticles. Particular studies aimed to overcome these limitations by confining the cobalt complex into a nanoporous solid, for example, by assembling it in the pore space of (NH_2_-)MIL-125(Ti)^37−40^ or embedding them as integral linker units in UU-100(Co) MOF.^41^ Although this significantly enhances the photocatalytic activity of the materials, a complex multistaged synthetic procedure is required, and concerns about the stability of the incorporated molecular cocatalysts have not yet been fully resolved. On the other hand, porous structures with single-site Co^II^ cocatalysts have been synthesized using a simple cobalt salt as a reactant via binding of Co^II^ to deprotonated μ_2_-O^–^ and μ_2_-O groups or coordination to μ_2_-oxide/(μ-carboxylate)_2_ groups of metal–oxo clusters of MOFs.^42−44^ These materials have demonstrated intriguing stability and performance in the photocatalytic hydrogenation and borylation of organic molecules. Nonetheless, the potential of this type of cobalt site for photocatalytic HER has not yet been investigated.

As such, we propose a novel, simple, and low-cost one-step procedure of cobalt(II) nitrate salt impregnation to introduce single-site Co^II^ into the structure of NH_2_-MIL-125(Ti), confirmed by XPS and XAS measurements. The photocatalytic performance of the synthesized Co@NH_2_-MIL-125(Ti) and pristine NH_2_-MIL-125(Ti) was assessed through HER experiments under visible (λ ≥ 385 nm) light exposure. The presence of the cobalt cocatalyst on the Ti–oxo cluster of the MOF led to almost 5 times increased photocatalytic activity compared to the pristine framework. Reuse of the photocatalysts was accompanied by structural changes in both NH_2_-MIL-125(Ti) and Co@NH_2_-MIL-125(Ti), leading to the formation of reactive proton reduction sites. Consequently, the H_2_ production was enhanced up to 45-fold for NH_2_-MIL-125(Ti) and up to 26-fold for Co@NH_2_-MIL-125(Ti).

## 2 Experimental Section

### 2.1 Materials and Reagents

Titanium isopropoxide (Ti(i-OPr)_4_, >97%, Sigma-Aldrich), 2-aminoterephthalic acid (98%, Sigma-Aldrich), *N,N*-dimethylformamide (DMF, 99.8%, extra dry over molecular sieve, Acros Organics), and methanol (99.8%, anhydrous, VWR) were used for synthesis of NH_2_-MIL-125(Ti). Cobalt(II) nitrate hexahydrate (Co(NO_3_)_2_·6H_2_O, ≥99.0%, Sigma-Aldrich) and acetone (≥99.5%, Sigma-Aldrich) were utilized for postsynthetic modification. For photocatalytic experiments, the mixture of acetonitrile (≥99.8%, anhydrous, Sigma-Aldrich), triethylamine (TEA, ≥99.0%, Sigma-Aldrich), and deionized water was prepared. All chemicals were used as received without further purification.

### 2.2 Synthesis of the Photocatalysts

#### 2.2.1 NH_2_-MIL-125(Ti)

The hydrothermal synthesis was performed according to Hendon et al.^12^ Briefly, 0.5 g of 2-aminoterephthalic acid, 16 mL of anhydrous DMF, and 4 mL of anhydrous methanol were mixed at room temperature in the air-free glovebox. After the linker was dissolved, 0.55 mL of titanium(IV) isopropoxide was added to the solution, and it was transferred to a PTFE/Teflon liner in a stainless-steel hydrothermal autoclave. The autoclave was transferred to an oven and heated for 72 h at 110 °C, followed by cooling at room temperature. Then, the mixture was filtered using a Büchner funnel and 0.45 μm nylon membrane filters. In a 100 mL glass bottle, the separated MOF catalyst was mixed with fresh DMF (≈40 mL) and heated overnight at 110 °C to remove excess linker. After vacuum filtration, the powder was rinsed with a small amount of methanol and immersed in fresh methanol (≈40 mL), followed by 6 h of heating at 80 °C. Then, the final product was separated from the mixture and dried in an oven for 3 h at 100 °C and further kept in a desiccator.

#### 2.2.2 Co@NH_2_-MIL-125(Ti)

For the preparation of Co@NH_2_-MIL-125(Ti), 150 mg of NH_2_-MIL-125(Ti) was suspended in 40 mL of acetone. Subsequently, 100 mg of cobalt(II) nitrate hexahydrate (1 wt %) was added, and the mixture was stirred for 3 h at room temperature. The MOF catalyst was extracted and rinsed with a small amount of acetone. The solids were washed by stirring overnight in acetonitrile (50 mL). After the subsequent filtration and rinsing with a small amount of fresh acetonitrile, the powder was dried at room temperature and further kept in a desiccator.

#### 2.2.3 NH_2_-MIL-125(Ti)-Act and Co@NH_2_-MIL-125(Ti)-Act

In order to preactivate synthesized photocatalysts, 90 mg of (Co@)NH_2_-MIL-125(Ti) was suspended in a mixture of 70.5 mL of acetonitrile, 14.1 mL of TEA, and 1.5 mL of water in a 100 mL glass bottle. The suspension was stirred and purged with a flow of Ar for 30 min to remove oxygen from the mixture. The bottle was further sealed, covered with aluminum foil to prevent light exposure, and stirred overnight. After the subsequent filtration and washing with fresh acetone, the final product was dried at room temperature and kept in a desiccator.

### 2.3 Characterization of Materials

#### 2.3.1 Powder X-ray Diffraction (PXRD)

PXRD patterns were acquired using a Bruker D8 Advance diffractometer with a Co Kα source (λ = 1.7889 Å, 35 kV and 40 mA) and a LynxEye position-sensitive detector. Diffractograms were collected with 2theta ranging from 5° to 50° at a scanning rate of 0.02° s^–1^, a motorized fixed-divergent slit 0.3°, and an exposure time of 0.7 s per step. Data refinement was performed in TOPAS *v.* 5.0.

#### 2.3.2 N_2_ Physisorption

N_2_ physisorption experiments were performed using a Micromeritics Tristar II 3020 at 77 K. Before sorption measurements, samples were dried overnight at 150 °C under N_2_ flow. The Brunauer–Emmett–Teller (BET) areas were calculated by using the BETSI computational tool^45^ with 11 data points in the 0.00–0.03 *p/p*_0_ range. The micropore volume of the photocatalysts, *V*_*micro*_, was derived using a *t*-plot method analysis of the N_2_ sorption isotherms.

#### 2.3.3 Transmission Electron Microscopy (TEM)

TEM measurements were performed by using a JEOL JEM-1400 Plus machine operating at an acceleration voltage of 120 kV. The samples were mounted on the carbon-coated copper grid.

#### 2.3.4 Diffuse Reflectance UV–vis (DRUV–vis)

DRUV–vis spectra were collected using a PerkinElmer Lambda 900 spectrophotometer with an integrating sphere in the range of 250–750 nm. BaSO_4_ was used as a white standard.

#### 2.3.5 Diffuse Reflectance Infrared Fourier Transform (DRIFT)

DRIFT spectra were recorded in the range of 4000–500 cm^–1^ using a Thermo Scientific Nicolet 8700 spectrometer equipped with a Praying Mantis high-temperature reaction chamber from Harrick. The samples were placed on top of the KBr filler material. Before measurements, the samples were dried at 150 °C in air, and data were collected at the same temperature. The spectral resolution was set to 2 cm^–1^ with 120 scan accumulation.

#### 2.3.6 Inductively Coupled Plasma Optical Emission Spectroscopy (ICP-OES)

ICP-OES analysis was used to determine the content of cocatalyst in the MOF by digesting as-synthesized and spent Co@NH_2_-MIL-125(Ti). The analysis was performed by Mikroanalytisches Laboratorium Kolbe.

#### 2.3.7 X-ray Photoelectron Spectroscopy (XPS)

XPS measurements were carried out to determine the chemical state and overall electronic structure of the elements of the synthesized MOFs. X-ray photoelectron spectra were recorded on a Thermo Scientific K-α spectrometer equipped with an aluminum monochromatic source (Al Kα, *h*ν = 1486.6 eV) and a 180° double-focusing hemispherical analyzer with a 128-channel detector.

#### 2.3.8 Thermogravimetric Analysis (TGA)

TGA was performed using a Mettler Toledo TGA/SDTA851e. The sample was heated from 20 to 800 °C at a heating rate of 10 °C/min under a synthetic air flow of 100 mL min^–1^.

#### 2.3.9 Time-Resolved Pump-Flow-Probe X-ray Absorption Spectroscopy (XAS)

Time-resolved pump-flow-probe XAS measurements were performed at the SuperXAS beamline of the Swiss Light Source at the Paul Scherrer Institute. The concept of the experiment setup has been described in detail elsewhere.^46^ In a typical measurement, 235 mg of Co@NH_2_-MIL-125(Ti) was suspended in a sample vial containing a mixture of 70 mL of acetonitrile, 14 mL of TEA, and 1.5 mL of deionized water and sonicated for 30 min. Before measurement, the sample jet chamber was purged with N_2_ for at least 15 min to deoxygenate the system. The sample solution was then pumped through a nozzle with a 1 mm diameter at a velocity of 4.32 m/s using a peristaltic pump. The absorbance of X-rays was measured after photoexcitation of the sample with a 400 nm laser with a 50 kHz repetition rate. The measurements were performed by following the pump-flow-probe method, where pumped data were collected for 5 s followed by unpumped data collection for another 5 s by switching the laser on and off, respectively. For each X-ray energy, the data collection was repeated at least 3 times. Transient spectra at different 70–1140 μs time delays were recorded by changing the distance between spatially separated laser and X-ray probe beams while keeping jet velocity constant at 4.32 m/s. Regular X-ray absorption near-edge structure (XANES) and extended X-ray absorption fine structure (EXAFS) spectra were collected using the same setup. The data were processed using an open-source X-ray Larch tool.^47,48^ EXAFS parameters refinement was done by fitting *k*^1^-, *k*^2^-, and *k*^3^-weighted oscillation FEFF paths. Further validation of the fit was done by evaluation using wavelet transform analysis.

The data concerning the characterization of the materials described in this work can be accessed at 4TU.ResearchData.^68^

### 2.4 Photocatalytic Experiments

In a typical experiment, 30 mg of sample was dispersed in a custom-made Pyrex-glass reactor with a mixture of 23.5:4.7:0.5 mL of acetonitrile/TEA/H_2_O, respectively. The suspension was stirred at a constant temperature (30 °C, maintained by the water jacket of the reactor) and purged with Ar flow (1.3 mL/min) for 30 min to remove air from the system. The evolved gases before and throughout the photocatalytic experiments were analyzed by withdrawing aliquots from the reactor headspace at different reaction times. The gases were carried by Ar flow and analyzed with a Chrompack CP 9001 gas chromatograph (GC) equipped with a TCD detector. Once the system was purged, Ar flow was reduced to 0.13 mL/min and the 500 W Xe/Hg lamp equipped with a 385 nm cutoff optical filter was turned on to start illumination. The photocatalytic reaction lasted at least 22.5 h. The stability of hydrogen production was also examined at a longer, 46.5 h, reaction time. During recyclability tests, photocatalysts were exposed to atmospheric conditions between each successive cycle, separated from the mixture, washed with fresh acetonitrile, and dried at 80 °C for 10 min before running the next cycle. The external quantum efficiency of the best-performing photocatalysts was calculated according to the previously reported procedure^18,37^ and is presented in the SI.

## 3 Results and Discussion

### 3.1 Characterization of Prepared Materials

The powder X-ray diffractograms of NH_2_-MIL-125(Ti) and Co@NH_2_-MIL-125(Ti) show sharp peaks that are consistent with the simulated crystal structure (Figure S1). No additional Bragg peaks of other phases or alterations in the refined lattice parameters are present.

N_2_ physisorption measurements of Co@NH_2_-MIL-125(Ti) showed a BET surface area of 1480 ± 19 m^2^ g^–1^, and a micropore volume of 0.55 cm^3^ g^–1^ (Figure S2), comparable to that of NH_2_-MIL-125(Ti) (1487 ± 13 m^2^ g^–1^, 0.56 cm^3^ g^–1^). The retention of adsorptive properties excludes the presence of large pore-blocking moieties and implies that the microporous structure is unaffected by the introduction of Co.^25,37,42^ TEM images of NH_2_-MIL-125(Ti) show well-defined crystallites with a size of approximately 200 nm that are retained after incorporation of Co without the formation of visible aggregates and oxidized nanoparticles within the MOF (Figure S3). ICP-OES analysis revealed that Co@NH_2_-MIL-125(Ti) contains approximately 1.5 wt % of Co, which corresponds to the incorporation of one Co in every third MOF cage.

**Figure 1:**
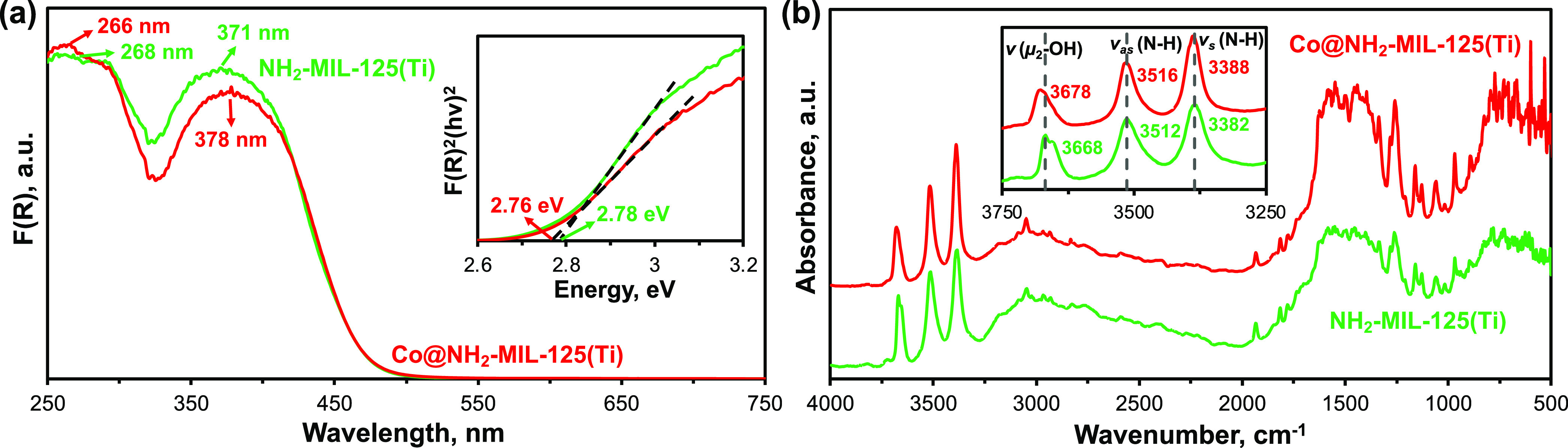
(a) Diffuse reflectance UV–vis spectra (inset corresponds to the Kubelka–Munk transformation of the spectra) and (b) DRIFT spectra of NH_2_-MIL-125(Ti) (green) and Co@NH_2_-MIL-125(Ti) (red) photocatalysts, respectively.

DRUV–vis spectra of the synthesized photocatalysts demonstrate two characteristic absorption bands (Figure 1a). The absorption band centered at the highest wavelength, around 375 nm, is typically assigned to the n– π* electron transition from the nitrogen atom in the amino groups of the linker (HOCO) to the 3*d*-orbital of Ti (LUCO) in the metal–oxo cluster of the MOF.^18,49,50^ Embedding of Co centers leads to only a marginal red shift of the HOCO-LUCO gap from 2.78 eV for NH_2_-MIL-125(Ti) to 2.76 eV for Co@NH_2_-MIL-125(Ti) which has been similarly reported with other incorporated transient metal centers.^25,34,51^ The observed insignificant changes most likely imply the remaining dominancy of the electronic transition from the π* orbital of the linker to the *d*-orbital of Ti in Co@NH_2_-MIL-125(Ti).

Interestingly, previously reported metal-doped NH_2_-MIL-125(Ti) MOFs showed a red shift of the N–H stretching vibrations in IR spectra, ascribed to the interaction of impregnated cocatalyst, such as an amine cobalt coordination complex, Cu or Pt or Au nanoparticles, with the amino groups of the ligand.^21,34,37^ In contrast, DRIFT spectra of dehydrated Co@NH_2_-MIL-125(Ti) in this study demonstrate a reproducible blue shift of the symmetric and asymmetric N–H stretching vibrations, Δ*v*_sym_= 6 cm^–1^ and Δ*v*_asym_= 4 cm^–1^, along with broadening and even more pronounced blue shift of the stretching vibrations of the bridging hydroxyls, μ_2_–OH groups, of the Ti–oxo clusters, Δ*v* = 10 cm^–1^ (Figure 1b). The blue shift indicates a contraction of the X–H bond (X = N, O) that could potentially be triggered by the presence of doped cobalt cations in the vicinity of the protons of the amino and hydroxyl groups.

**Figure 2:**
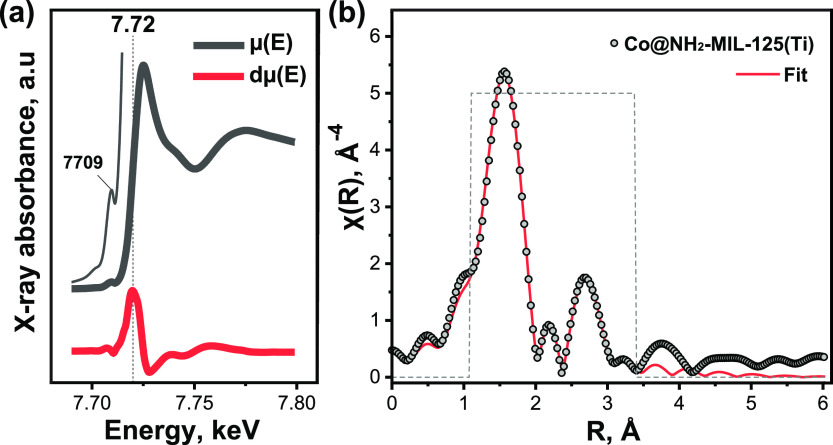
(a) Co K-edge XANES spectrum (gray) and its first derivative (red), and (b) EXAFS R-space spectrum of Co@NH_2_-MIL-125(Ti).

High-resolution XPS spectra of both Co@NH_2_-MIL-125(Ti) and NH_2_-MIL-125(Ti) show the expected Ti^IV^ state^52^ and that most of the cobalt in Co@NH_2_-MIL-125(Ti) is present as Co^II^ (Figure S4).^42,53^ The Co K-edge XANES spectrum (Figure 2a) features a low-intensity pre-edge peak at around 7709 eV typical of the electric-dipole-forbidden 1*s* → 3*d* electronic transition of Co^II^ enabled by mixing 3dz–4pz orbitals, which occurs upon (axial) distortion of the octahedral ligand environment. The position of the edge was found from the first derivative of Co K-edge XANES spectrum at 7720 eV, notably shifted from the edge position of the Co(0) foil reference −7709 eV (Figure S5a), and corresponds to the oxidation state +2.^40,54−56^

The local coordination environment of Co was further studied with EXAFS. The first coordination shell is featured in the intense scattering at around 1.5 Å in the R space (Figure 2b and Table S1). Further analysis using wavelet transform (Figure S5b) revealed the presence of low-intensity scatterers at around 2.7 Å. The best fit was achieved for a model with O at 2.06 Å and O (or N) at 2.34 Å with coordination numbers of 5.1 and 1.3, respectively. This corresponds well to the distorted octahedral coordination of Co atoms. The second coordination shell is formed by Ti atoms at 3.1 Å. The combination of the DRIFT and EXAFS results suggests that Co likely forms monatomic sites stabilized on (by) the Ti–oxo cluster. In this case, Co centers can be chelated by two bridging hydroxo (μ_2_–OH) and two bridging oxo (μ_2_–O) groups^42^ or coordinated to μ_2_-(hydr)oxide/μ-carboxylates of the metal cluster of the MOF.^40,42^ The optimized Co–Ti coordination number suggests that the majority of Co centers are not bound to oxygens involved in the μ_2_-(hydr)oxo bridges between Ti atoms (Figure S6a). Instead, the presence of the cocatalyst coordinated to single Ti atoms via μ-carboxylate groups (Figure S6b) is more consistent with our fitting of the EXAFS profile.

### 3.2 Photocatalytic H_2_ Evolution Experiments

**Figure 3:**
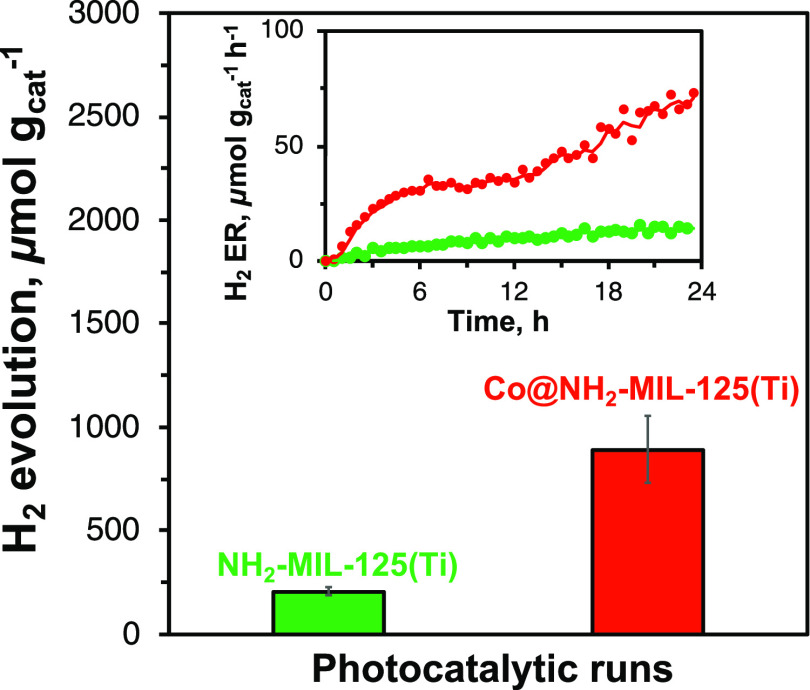
Accumulated amount of evolved hydrogen after 22.5 h of irradiation and H_2_ evolution rates (inset) over the photocatalysts. Experimental data of the H_2_ evolution rate are represented by closed circles with a moving average solid line.

The photocatalytic hydrogen evolution rates of NH_2_-MIL-125(Ti) and Co@NH_2_-MIL-125(Ti) are shown in Figure 3. After 22.5 h of light exposure, around 200 μmol g_cat_^–1^ and 900 μmol g_cat_^–1^ of H_2_ was evolved for NH_2_-MIL-125(Ti) and Co@NH_2_-MIL-125(Ti), respectively (Figure 3). The H_2_ evolution of our Co@NH_2_-MIL-125(Ti) is higher than that reported for nonactivated NH_2_-MIL-125(Ti) with a cobalt coordination complex derived from cobaloxime (2.7 wt % of Co), reaching around 670 μmol g_cat_^–1^ under similar conditions.^37,40^ In addition, both samples demonstrate a gradual increase in the H_2_ evolution rate over time (inset in Figure 3), indicating an activation period, commonly observed for NH_2_-MIL-125(Ti)-derived photocatalysts, and no apparent deactivation stage, typically accompanied by a decrease in H_2_ production rates.^18,21,23,37^

**Figure 4:**
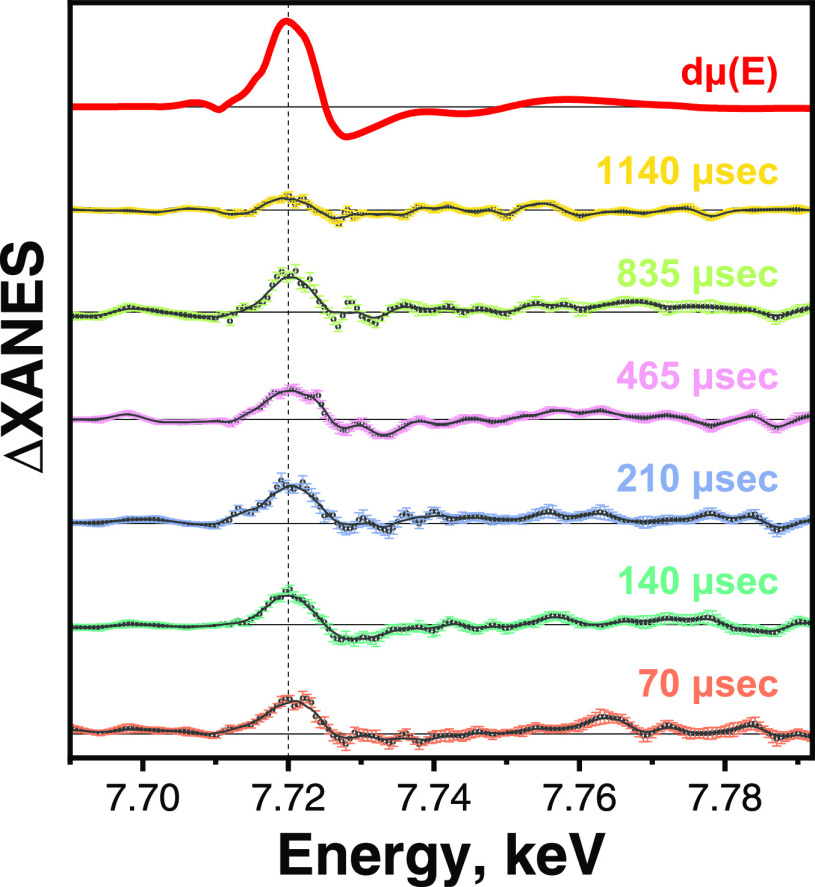
First derivative of Co K-edge XANES spectrum (red) and differential transient Co K-edge XANES spectra at 70–1140 μs time delays for Co@NH_2_-MIL-125(Ti). Measured data are represented by open circles, and lines are the smoothed spectra (adjacent averaging with a window of 5 data points with corresponding standard error of the mean).

To probe the oxidation state of Co during the photocatalytic reaction and unravel its role in the reaction mechanism, we performed a pump-flow-probe experiment to obtain transient Co K-edge XANES spectra. Co@NH_2_-MIL-125(Ti) was suspended in the photocatalytic reaction solution and excited with 400 nm femtosecond pulsed light running at a high repetition rate (50 kHz). The transient XANES spectra show a significant increase in X-ray absorption around 7720 eV with a lifetime extending from the microsecond to the millisecond range (Figure 4). In some systems, an additional decrease in white line intensity around 7730 eV (a strong negative peak in the transient signal) was ascribed to a four-coordinated Co^I^ center,^57,58^ while in systems without additional ligand loss, the strong negative features were not observed.^59^ Consequently, the presence of only a positive peak at around 7730 eV (Figure 4) suggests a coordination number higher than four for the Co^I^ center. Besides, such shifts of absorption edges to lower energies (7720 eV) are typical for the reduction of the Co center in molecular hydrogen-evolving catalysts.^57−60^ The formation of a Co^I^ intermediate was also suggested in the previously discussed amine cobalt coordination complex embedded in NH_2_-MIL-125(Ti) based on *operando* light “on”–light “off” XANES experiments.^40^

Previous electron paramagnetic resonance studies of NH_2_-MIL-125(Ti) with introduced Co species^25,40^ demonstrated the presence of Ti^III^ sites upon photoexcitation and their likely involvement in the charge-transfer processes with the embedded cocatalyst. Moreover, the inclusion of Co species into NH_2_–UiO-66(Zr) and NH_2_-MIL-53(Al) with Zr- and Al-based metal nodes did not yield any improvements in the H_2_ evolution. This makes the sole role of Co species in driving the photocatalytic reaction highly unlikely and corroborates the involvement of Ti nodes in electron transfer.^40^ Therefore, considering that the intensity of the transient signal does not increase within the measured time window, likely electron transfer from Ti^III^ leading to conversion of Co^II^ to Co^I^ might take place on a time scale shorter than 70 μs. Besides, the catalytic conversion of Co^I^ and H^+^, originating from water molecules and/or from protonated TEA base,^37^ to the expected Co^III^–H species^36,61,62^ is much slower. Nevertheless, the much lower photocatalytic activity of pristine NH_2_-MIL-125(Ti) versus Co@NH_2_-MIL-125(Ti) implies that proton reduction via the direct electron transfer from Ti^III^ is even slower.

### 3.3 Reusability Tests

**Figure 5:**
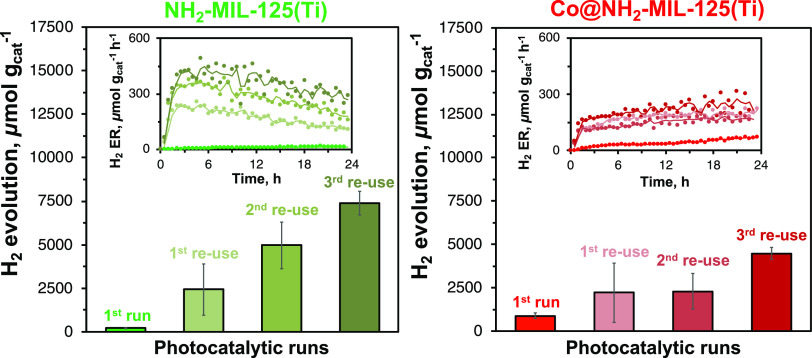
Accumulated amount of evolved hydrogen after 22.5 h of irradiation and H_2_ evolution rates (inset) over (Co@)NH_2_-MIL-125(Ti) and one to three times reused photocatalysts. Experimental data of the H_2_ evolution rate are represented by closed circles with a moving average solid line.

A series of recycling experiments was performed to assess the stability of the synthesized catalysts. Unexpectedly, each successive photocatalytic run leads to a stepwise increase of total evolved H_2_ for both studied MOFs (Figure 5). However, this is less pronounced for Co@NH_2_-MIL-125(Ti) compared to NH_2_-MIL-125(Ti). After four sequential cycles, the amount of produced H_2_ reached up to 8050 μmol g_cat_^–1^ and 4810 μmol g_cat_^–1^ during the third reuse of NH_2_-MIL-125(Ti) and Co@NH_2_-MIL-125(Ti), respectively, indicating a 40- and 24-fold increase, when compared to pristine NH_2_-MIL-125(Ti).

Notably, except for the first photocatalytic run, during each run, both photocatalysts acquire a distinctive increase in the hydrogen evolution rate during the first 90 min of light exposure (insets in Figure 5). For NH_2_-MIL-125(Ti), however, this is followed by a gradual decrease in the H_2_ evolution rate during each catalytic run, signifying a gradual deactivation of the photocatalyst. In contrast, the photoactivity of Co@NH_2_-MIL-125(Ti) remains stable or even slightly increases after 90 min without any signs of deactivation. Finally, at the end of the third recycling run, both photocatalysts have a similar hydrogen evolution rate: 300 μmol g_cat_^–1^ h^–1^ and 250 μmol g_cat_^–1^ h^–1^ for NH_2_-MIL-125(Ti) and Co@NH_2_-MIL-125(Ti), respectively.

The initial increase in the photocatalytic activity of the reused catalysts begs the question of which conditions are needed for this activation. Previous reports discuss the preactivating of NH_2_-MIL-125(Ti) or its modifications before the photocatalytic reaction, for example, by introducing a washing protocol^37^ or a light-induced thermal treatment^15^ to enhance the overall photocatalytic activity of the MOFs. Thus, we suspended pristine NH_2_-MIL-125(Ti) and Co@NH_2_-MIL-125(Ti) into the reactant mixture without light exposure, resulting in NH_2_-MIL-125(Ti)-Act and Co@NH_2_-MIL-125(Ti)-Act, respectively.

**Figure 6:**
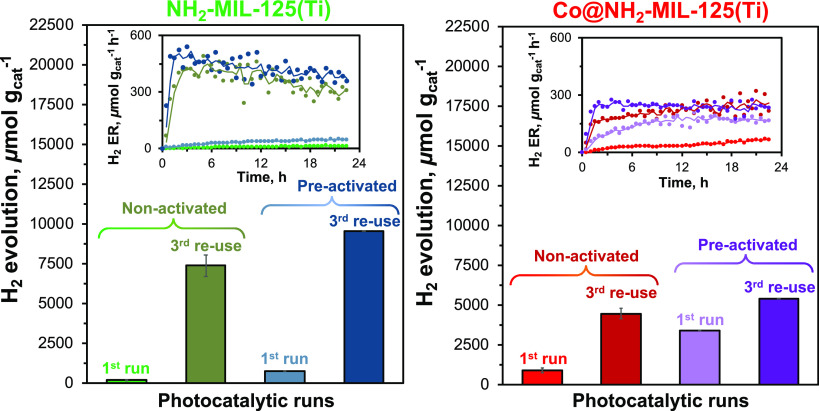
Accumulated amount of evolved hydrogen after 22.5 h of irradiation and H_2_ evolution rates (inset) over (Co@)NH_2_-MIL-125(Ti), preactivated, and three times reused photocatalysts. Experimental data of the H_2_ evolution rate are represented by closed circles with a moving average solid line.

In Figures 6 and S8, the photocatalytic activity of preactivated samples and nonactivated photocatalysts are shown. For Co@NH_2_-MIL-125(Ti)-Act, a more than 16-fold increase in the amount of produced H_2_ and a distinctive rise in the H_2_ evolution rate are observed during the first photocatalytic run, while NH_2_-MIL-125(Ti)-Act shows only a 4-fold increase after the first run. Therefore, immersion of Co@NH_2_-MIL-125(Ti) in the synthetic solution plays a predominant role in the activation, while for NH_2_-MIL-125(Ti) the presence of light is necessary to achieve a proper activation.^15^

**Figure 7:**
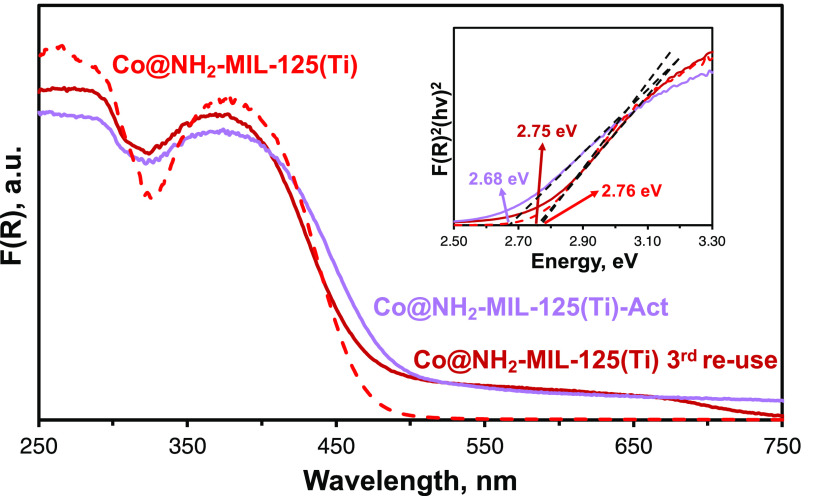
DRUV–vis spectra of Co@NH_2_-MIL-125(Ti), preactivated, and reused photocatalysts.

The DRUV–vis spectrum of NH_2_-MIL-125(Ti)-Act only shows minor changes compared to NH_2_-MIL-125(Ti) (Figure S9), while spectra of Co@NH_2_-MIL-125(Ti)-Act directly after activation and Co@NH_2_-MIL-125(Ti) after the fourth photocatalytic run are significantly different from those of pristine Co@NH_2_-MIL-125(Ti). A pronounced broad absorbance ranging from 500 to 800 nm appeared (Figure 7), suggesting the emergence of a new HOCO/LUCO gap with lower energy, potentially associated with *d*–*d* transitions of the Co atoms. The latter can be caused by the restructuring of the Co^II^ sites in the reaction medium, including triethylamine, which improves the absorbance of visible light of the MOF or enhances the proton reduction activity of the Co sites.

However, after three times recycling, both preactivated NH_2_-MIL-125(Ti)-Act and Co@NH_2_-MIL-125(Ti)-Act and non-preactivated NH_2_-MIL-125(Ti) and Co@NH_2_-MIL-125(Ti) catalysts demonstrate a similar H_2_ evolution rate (Figure 6). Again, a decrease in photocatalytic activity with time is observed for the recycled NH_2_-MIL-125(Ti)-Act, while not for the recycled Co@NH_2_-MIL-125(Ti)-Act materials. For a prolonged reaction time of 46.5 h, we observe a stable hydrogen evolution for both three times reused Co@NH_2_-MIL-125(Ti)-Act and NH_2_-MIL-125(Ti)-Act from 24 h onward, around 230 and 370 μmol g^–1^ h^–1^, respectively (Figure S10). The external quantum efficiency (EQE) for these samples is ca. 0.14 and 0.24%, respectively, and only 0.03% for pristine NH_2_-MIL-125(Ti).^18^

**Figure 8:**
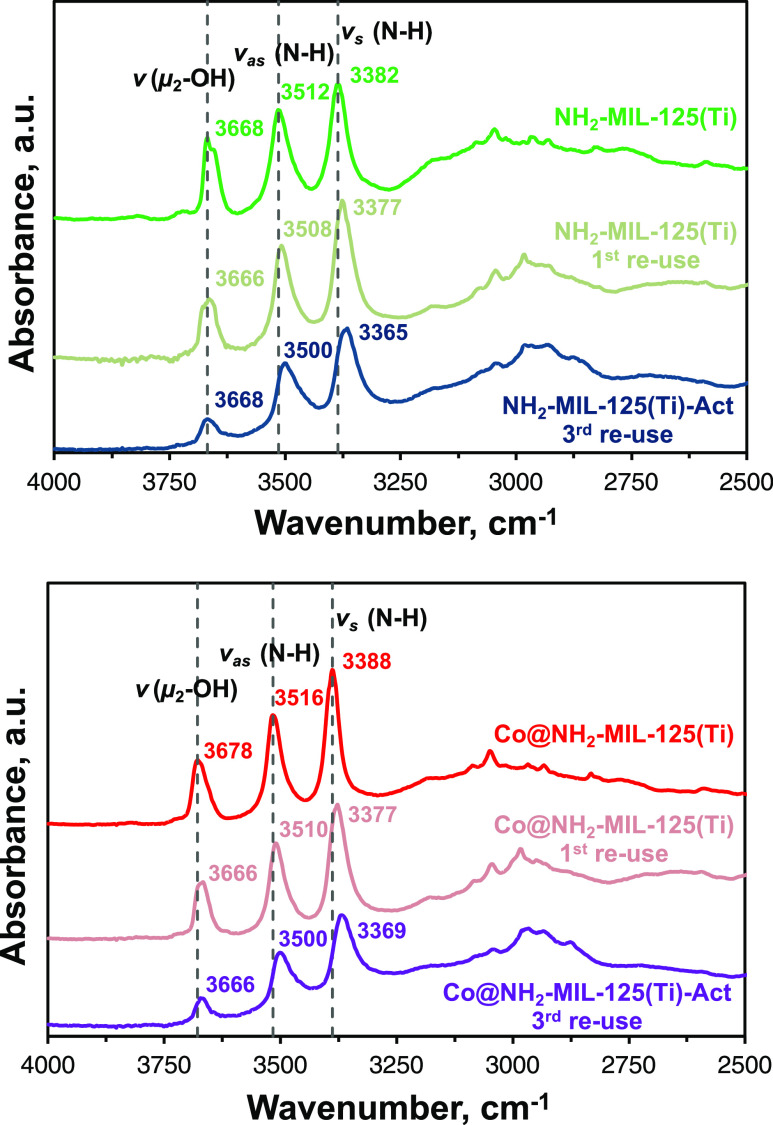
DRIFT spectra of (Co@)NH_2_-MIL-125(Ti), preactivated, and reused photocatalysts.

The recorded DRIFT spectra of all reused (Figures 8 and S11a) and preactivated photocatalysts (Figure S11b) show an increased intensity at 2980 cm^–1^ that can be ascribed to asymmetric and symmetric C–H stretching modes of adsorbed TEA molecules on the MOFs.^63,64^ The presence of adsorbed TEA or its decomposition products is also supported by TGA profiles of both three times reused photocatalysts (Figure S12) with a continuous weight loss up to 250 °C. The increasing number of stretching vibrations in the 1200–1050 cm^–1^ region after several photocatalytic runs might indicate the formation and accumulation of unidentified organic species in the pores of the framework, such as partially oxidized species or condensation products of TEA.^65,66^ Furthermore, three times reused NH_2_-MIL-125(Ti) and Co@NH_2_-MIL-125(Ti) show a distinguishable 64% and 44% decrease, respectively, of μ_2_–OH area relative to *v*_sym_ (N–H) compared to their pristine counterparts. This likely signifies proton loss from titanol groups in the inorganic cluster: Ti–OH → Ti–O^–^. In addition, the previously observed blue shift of amino and titanol stretching vibrations (Figure 1b) of Co@NH_2_-MIL-125(Ti) compared to NH_2_-MIL-125(Ti) has been significantly diminished after three photocatalytic cycles.

Analysis of the PXRD pattern of three times reused NH_2_-MIL-125(Ti) reveals broadening and a decrease in intensity of all of the reflections (Figure S13). In contrast to the systems with triethanolamine as a sacrificial agent, where degradation of the catalyst occurs after 9 h of visible light photocatalysis,^15^ reused NH_2_-MIL-125(Ti) in the present work shows a relatively minor loss of crystallinity after at least four photocatalytic cycles, while spent Co@NH_2_-MIL-125(Ti) fully retains its original crystallinity. The XPS data (Figure S14) of NH_2_-MIL-125(Ti) indicate a noticeable decrease in the N/Ti ratio already after the first reuse (Table S2), which can signify a partial linker elimination, while only insignificant changes are observed for reused Co@NH_2_-MIL-125(Ti). Furthermore, a distinguishable change in the metal-to-linker ratio calculated from TGA profiles (Figure S12) confirms up to 18% removal of organic linkers for NH_2_-MIL-125(Ti) after the third reuse, while this is only 9% for Co@NH_2_-MIL-125(Ti). It was previously reported that photothermal treatment of NH_2_-MIL-125(Ti) in the presence of triethanolamine leads to consequent linker dissociation and further framework deterioration.^15,67^ The incorporated cobalt in Co@NH_2_-MIL-125(Ti) seems to hinder linker elimination and defect formation in the framework. Besides, ICP-OES analysis of reused Co@NH_2_-MIL-125(Ti) revealed only negligible loss of Co from the catalyst (Table S3).

### 3.4 Mechanism of Visible-Light-Driven H_2_ Evolution over NH_2_-MIL-125(Ti) and Co@NH_2_-MIL-125(Ti)

**Figure 9:**
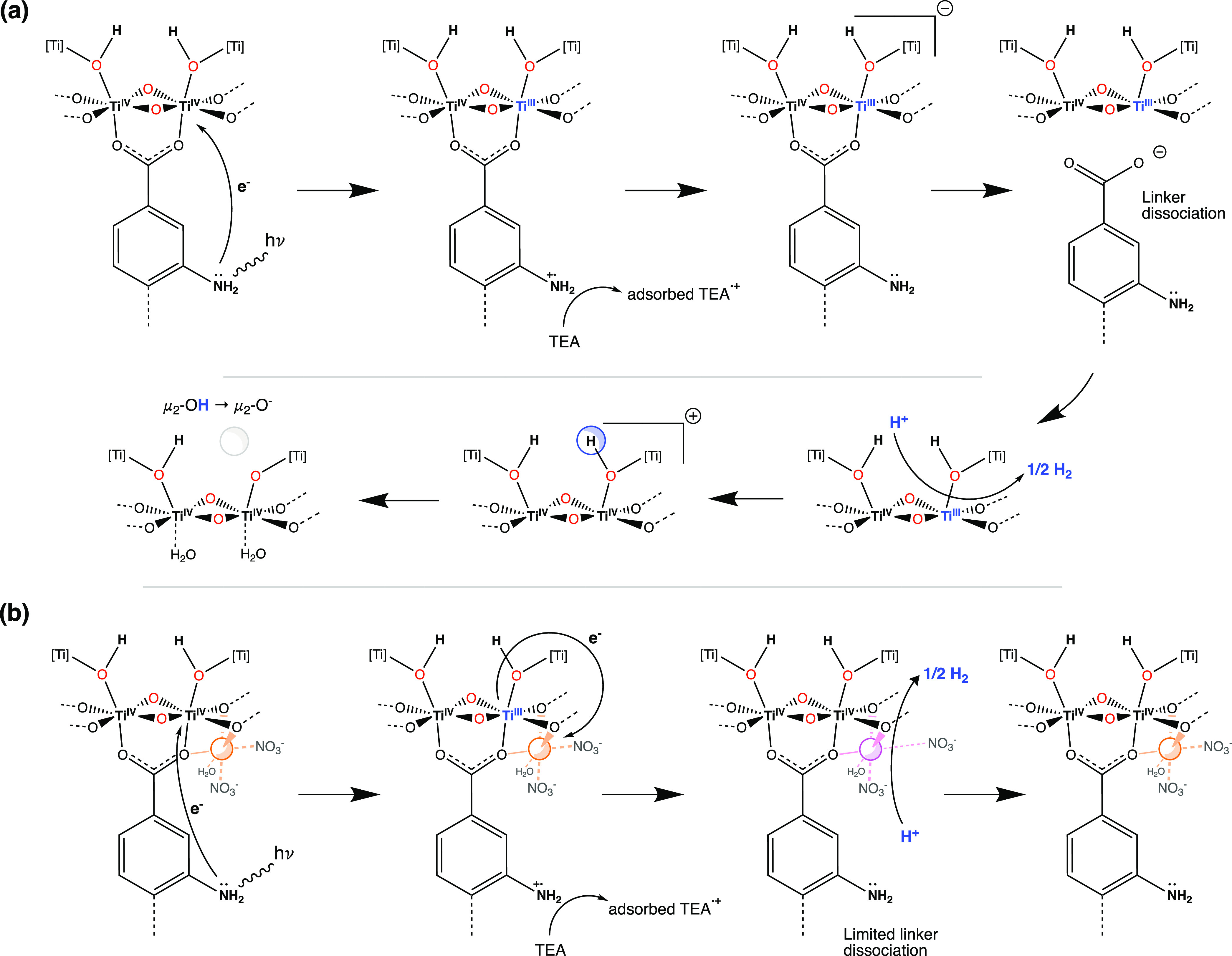
Schematic representation of the mechanism of the H_2_ evolution reaction over (a) NH_2_-MIL-125(Ti) and (b) Co@NH_2_-MIL-125(Ti).

To rationalize all of the discussed observations, we propose the following mechanism for the light-driven hydrogen evolution on our catalysts (Figure 9). Absorption of the light by NH_2_-MIL-125(Ti) leads to charge transfer of a photoexcited electron on the organic linker of the MOF to the Ti–oxo cluster through a LMCT mechanism, leading to the formation of a positively charged radical –NH_2_^+•^ and Ti^III^.^18,49^ Although the generated holes can be effectively quenched by the sacrificial electron donor, triethylamine, the lack of active proton reduction sites results in poor H_2_ production (Figure 3).^15^ Despite this, the photocatalytic activity of NH_2_-MIL-125(Ti) increases upon recycling the spent catalyst. We hypothesize that recycling leads to notable linker elimination from the framework, which would allow for the negatively charged Ti^III^-containing cluster to become neutral.^15^ After proton reduction and return to its original Ti^IV^ state, a cluster with a missing linker would be positively charged, which can in principle be balanced by a proton leaving μ_2_–OH, reducing the group to μ_2_–O^–^.^50^ The latter is in line with the IR data (Figure 8) showing the strong decrease in the amount of hydroxyls during subsequent photocatalytic cycles, and we hypothesize that this is predominantly due to proton removal. Detachment of the linker from the inorganic cluster also implies the formation of coordinatively unsaturated Ti sites or their coordination to less strongly bonded water molecules. In either case, these sites are expected to show higher reactivity as proton reduction centers.^14,15^

The photocatalytic activity of pristine Co@NH_2_-MIL-125(Ti) is significantly higher than that of NH_2_-MIL-125(Ti). The differential transient XANES spectra (Figure 4) indicate a mechanism via a Co^I^ intermediate (highly likely via electron transfer from Ti^III^ to Co^II^), acting as a proton reduction site. We suppose this charge transfer can also be involved in the charge compensation mechanism, via promoting the return of Ti^III^ to Ti^IV^ and thus reducing the negative charge of the photoexcited inorganic cluster and limiting linker elimination from the framework (Figure S12 and Table S2). The further stepwise increase in photocatalytic activity upon recycling the photocatalyst is likely attributable to (i) the activation via restructuring of Co^II^ via immersion in the reaction medium (Figure 7) and (ii) missing linker defects forming additional coordinatively unsaturated titanium sites as proton reduction sites.^42^ The latter is similar to NH_2_-MIL-125(Ti), but the loss of bridged hydroxyls (Figure 8) and organic linkers (Figure S12) is much smaller. Overall, the presence of the Co^II^ cocatalyst on the Ti–oxo cluster restrains linker removal, preserving the original crystallinity of Co@NH_2_-MIL-125(Ti) under photocatalytic conditions, and leads to a stable H_2_ production even at a prolonged reaction time (Figures S10 and S13).

## 4 Conclusions

In summary, we have enhanced the visible light photocatalytic hydrogen evolution activity of NH_2_-MIL-125(Ti) by creating reactive proton reduction sites in the framework. The present work demonstrates an original approach to synthesize NH_2_-MIL-125(Ti) with Co^II^ cocatalytic single sites, residing on the Ti–oxo cluster of the MOF, via a simple impregnation with Co(NO_3_)_2_. Pump-flow-probe XANES spectroscopy uncovered transient Co^I^ species, likely via electron transfer from photoexcited Ti^III^ to Co^II^, that prolonged the lifetime of the charge separation state and enhanced the photocatalytic activity of the MOF for hydrogen evolution. Furthermore, we found that the photocatalytic reaction with triethylamine as a sacrificial electron donor activates both photocatalysts; after reusing the photocatalyst three times, NH_2_-MIL-125(Ti) and Co@NH_2_-MIL-125(Ti) demonstrated up to a 45- and 26-fold enhancement of H_2_ production compared to pristine NH_2_-MIL-125(Ti), respectively.

For NH_2_-MIL-125(Ti), the drastically higher photoactivity is a result of undergoing linker elimination from the framework during photocatalysis, leading to the formation of coordinatively unsaturated Ti sites, which act as proton reduction centers. On the other hand, reusing Co@NH_2_-MIL-125(Ti) results in less-pronounced linker removal, while the largest part of the photocatalytic enhancement originates from the incorporated and activated Co^II^ cocatalyst sites. Moreover, embedding Co sites results in highly stable Co@NH_2_-MIL-125(Ti) with more persistent H_2_ production than NH_2_-MIL-125(Ti), even at a prolonged reaction time. Further experimental and computational studies on modulating Ti–oxo clusters with incorporated metal single-site centers (e.g., Co, Cu, Ni) can deepen our understanding of their incorporation effects on the electronic structure, photoactivity properties, and structural stability of the MOFs. Overall, this work provides a novel strategy for introducing single-site cocatalysts into the MOF complemented with insights into the synthesis, activation, and development of stable and highly efficient visible-light-responsive MOF-based photocatalysts.

## Data Availability

The data concerning the characterization of the materials described in this work can be accessed and used by others for further studies at 4TU.ResearchData at https://doi.org/10.4121/e3fdc43f-4d54-4f15-9c1f-f68c6b23fceb.

## Supporting Information Available

PXRD patterns; N_2_ sorption isotherms; TEM images; high-resolution N 1s, Ti 2p, and Co 2p spectra; DRUV–vis and DRIFT spectra; TGA profiles of photocatalysts; the spectrum of the 500 W Xe/Hg light source; XAS data analysis; external quantum efficiency calculations; and additional experimental data on the amount of evolved H_2_ after 22.5 and 46.5 h over photocatalysts (PDF)
