# Supplementary material for: Promoting Photocatalytic Activity of NH2-MIL-125(Ti) for H2 Evolution Reaction through Creation of TiIII- and CoI-Based Proton Reduction Sites
Source: ACS Appl Mater Interfaces. 2023 Nov 15;15(47):54590–601. doi: 10.1021/acsami.3c15490 (PMC10694822; doi:10.1021/acsami.3c15490)
Supplement: Supplementary file 1 — am3c15490_si_001.pdf [file am3c15490_si_001.pdf]

Supporting Information

Promoting photocatalytic activity of NH_2-MIL-125(Ti) for H_2 evolution reaction through creation of Ti^{III}- and Co^{I}-based proton reduction sites

Vitalii Kavun,[*a] Evgeny Uslamin,[b] Bart van der Linden,[b] Stefano Canossa,[c] Andrey Goryachev,[b] Emma E. Bos,[b] Jara Garcia Santaclara,[b] Grigory Smolentsev,[d] Eveliina Repo,[a] Monique A. van der Veen[*b]

[a]*Department of Separation Science, LUT University, FI-53850, Lappeenranta, Finland.*

[b]*Department of Chemical Engineering, Delft University of Technology, 2629 HZ, Delft, The Netherlands.*

[c]*Department of Nanochemistry, Max Planck Institute for Solid State Research, 70569, Stuttgart, Germany.*

[d]*Paul-Scherrer Institute, CH-5232, Villigen PSI, Switzerland.*

[*]*Corresponding authors: vitalii.kavun@lut.fi, vitalii.kavun@gmail.com, m.a.vanderveen@tudelft.nl*

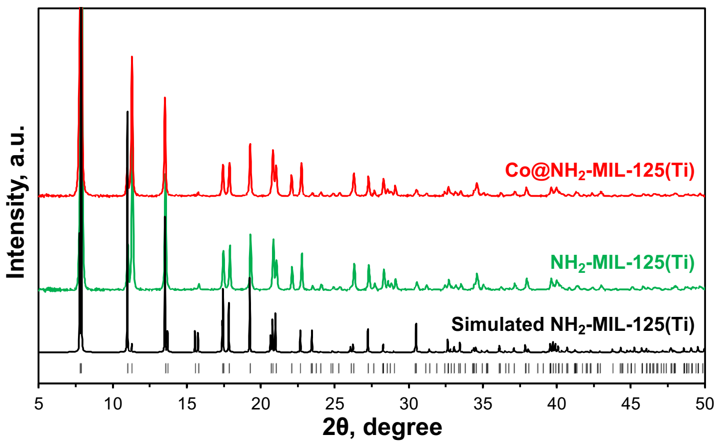

Figure S1. PXRD patterns of photocatalysts. Refined lattice constant: $a = b = 18.664(1)$ Å, $c = 18.129(1)$ Å for NH_2-MIL-125(Ti) and $a = b = 18.648\ (1)$ Å, $c = 18.113(1)$ Å for Co@NH_2-MIL-125(Ti). Differences in peak intensity of the MOFs can be due to changes in pore content, molecular conformations, and crystal morphology.

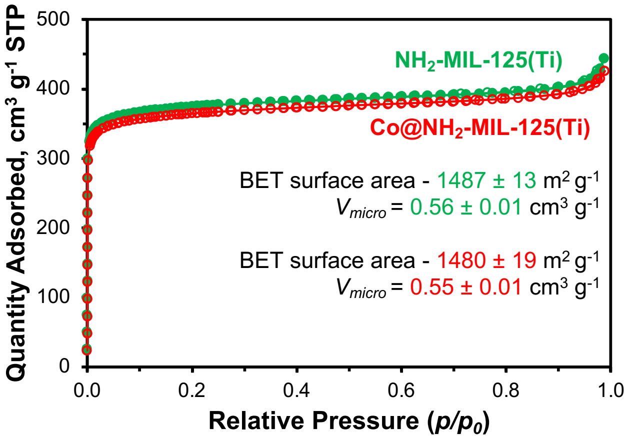

Figure S2. Nitrogen sorption isotherms of photocatalysts at 77 K. Closed and open circles denote adsorption and desorption datapoints, respectively. Small changes in the N_2 sorption results corroborate the single-site nature of the incorporated Co-atoms, and might also result from the extra step of the post-modification of NH_2-MIL-125(Ti) in acetone, removing the traces of DMF and/or unreacted linkers more thoroughly from the MOF pores.

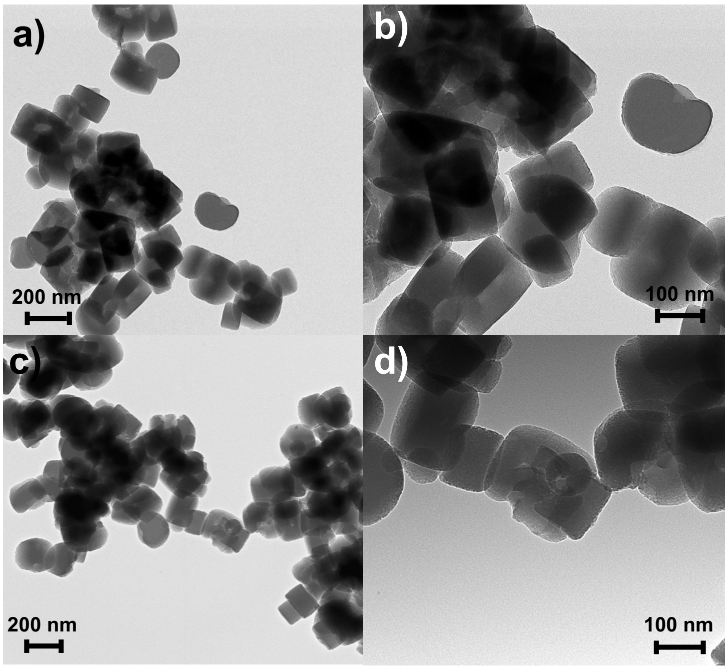

Figure S3. TEM images of pristine NH_2-MIL-125(Ti) and Co@NH_2-MIL-125(Ti) at 200 nm (a and c) and 100 nm (b and d), respectively.

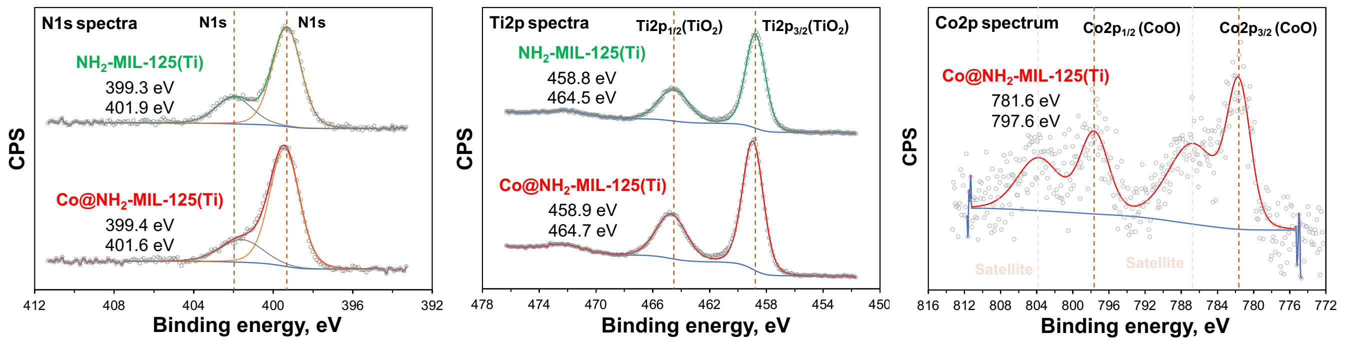

Figure S4. High resolution N1s, Ti2p and Co2p spectra of NH$_2$-MIL-125(Ti) and Co@NH$_2$-MIL-125(Ti) photocatalysts.

N1s spectra of NH$_2$-MIL-125(Ti) and Co@NH$_2$-MIL-125(Ti) samples are composed of two distinct peaks with maxima at 399.4 ± 0.1 eV and 401.8 ± 0.2 eV, assigned to -NH$_2$ groups and positively charged nitrogen species (R-NH$_3^+$, R-NH$^+$-) in *sp*^{2} C environment of organic linker.[1–4] Two peaks at 458.7 ± 0.4 eV (Ti2p$_{3/2}$) and 464.5 ± 0.3 eV (Ti2p$_{1/2}$) with shake-up lines appearance on the high resolution XPS spectra of both photocatalysts correspond to Ti^{IV} state in the MOF structure. Slight variation in Ti2p binding energies (BE) is attributed to the differential charging of Ti-oxo clusters within the MOF structure. Co2p$_{3/2}$ and Co2p$_{1/2}$ BE values of 781.6 and 797.6 ± 0.1 eV with shake-up features suggest major presence of Co^{II} sites in Co@NH$_2$-MIL-125(Ti).[5]

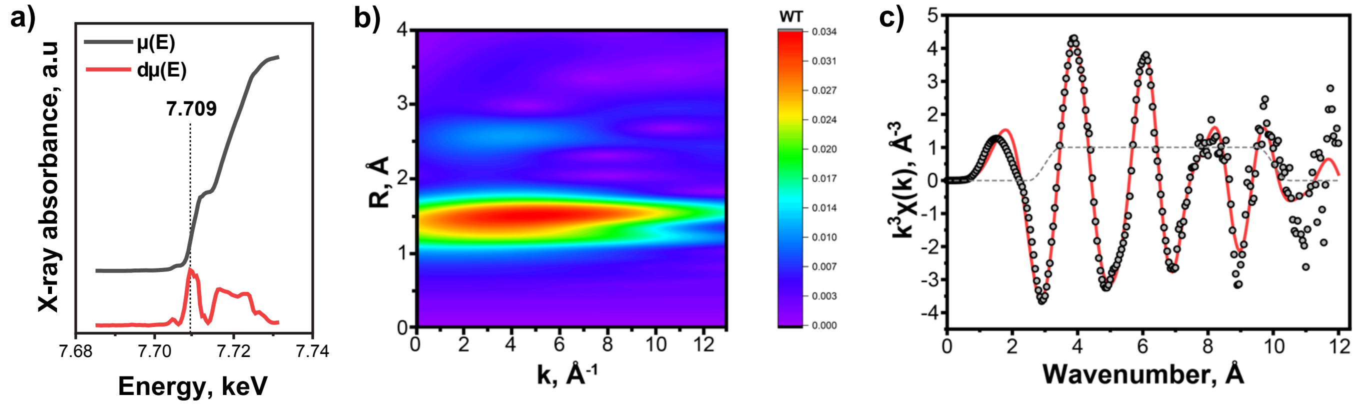

Figure S5. (a) Co K-edge XANES spectrum of Co foil (grey) and its first derivative (red), (b) Wavelet transformed and (c) k^3-weighted representation of EXAFS data for Co@NH$_2$-MIL-125(Ti). The fit is shown as a red line on the k^3-weighted spectrum.

Table S1. Summary of EXAFS fitting parameters.

Path	Distance (Å)	Coordination number (*N*)	Mean-square disorder in the distribution of interatomic distances (σ^2)	Energy shift (E_0)	*R* factor
Co – O	2.06	5.1 ± 0.2	0.01		
Co – O	2.35	1.3 ± 0.3	0.01	0.7 ± 0.3	0.001
Co – Ti	3.07	1.1 ± 0.1	0.01		

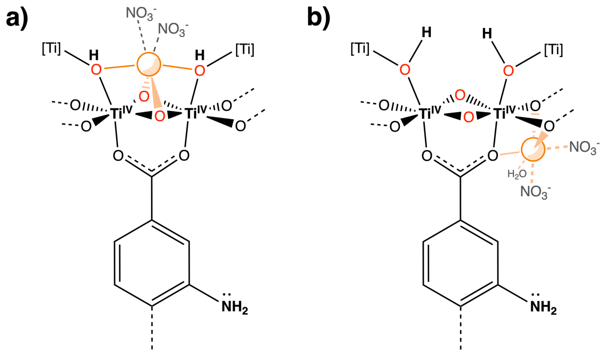

Figure S6. Schematic representation of two (a and b) possible Co coordination modes in Co@NH$_2$-MIL-125(Ti).

External quantum efficiency (EQE)

External quantum efficiencies (EQE) of photocatalysts were calculated according to the following equation:

$$\Phi = \frac{E(t)}{P(t)} \qquad (1)$$

where $E(t)$ is a number of electrons carrying out a redox reaction per unit of time; $P(t)$ is a photon flux per unit of time.

The light source intensity of 500 W Xe/Hg lamp used in this work is determined using an AvaSpec-3648-2-USB2 (Avantes, the Netherlands). The recorded spectrum presented on Figure S7 demonstrates incident photon flux as a function of wavelength that is obtained with 385 nm cut-off filter and at the distance of 5.0 cm from the light source (same as for the photocatalytic experiment).

The curve was integrated to derive the total integral lamp intensity at the range from 350 nm (where flux goes to 0) to 455 nm (considering HOMO-LUMO gap of the photocatalysts), yielding 6427.6 μmol(photons) s^{-1} m^{-2}.

Considering 3.14 cm^2 illuminated area (spot size at 5.0 cm distance), the photon flux $P(t)$ = 6427.6 μmol(photons) s^{-1} m^{-2} • 0.000314 m^2 = 2.02 μmol(photons) s^{-1} or 7269.4 μmol(photons) h^{-1}.

For each hydrogen molecule there are 2 electrons required for its production. Taking the H_2 evolution rate of three times re-used NH_2-MIL-125(Ti) photocatalyst as 8.7 μmol(H_2) h^{-1}, thus, a number of utilized electrons, $E(t)$, would be 17.4 μmol(electrons) h^{-1}. Therefore, EQE of three times re-used NH_2-MIL-125(Ti) photocatalyst was calculated using Equation 1:

$$\Phi = \frac{E(t)}{P(t)} = \frac{17.4\ \mu\text{mol(electrons)}\ h^{-1}}{7269.4\ \mu\text{mol(photons)}\ h^{-1}} = 0.0024 = 0.24\%$$

EQEs for other photocatalysts were derived in the same manner.

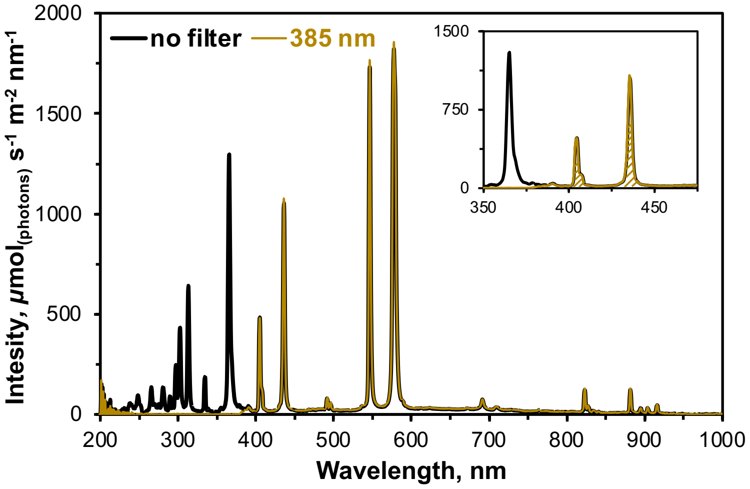

Figure S7. Spectrum of the 500W Xe/Hg light source. Light without filter (black) and with 385 nm cut-off filter (brown). The integral area at 350-455 nm range is shown filled in the inset.

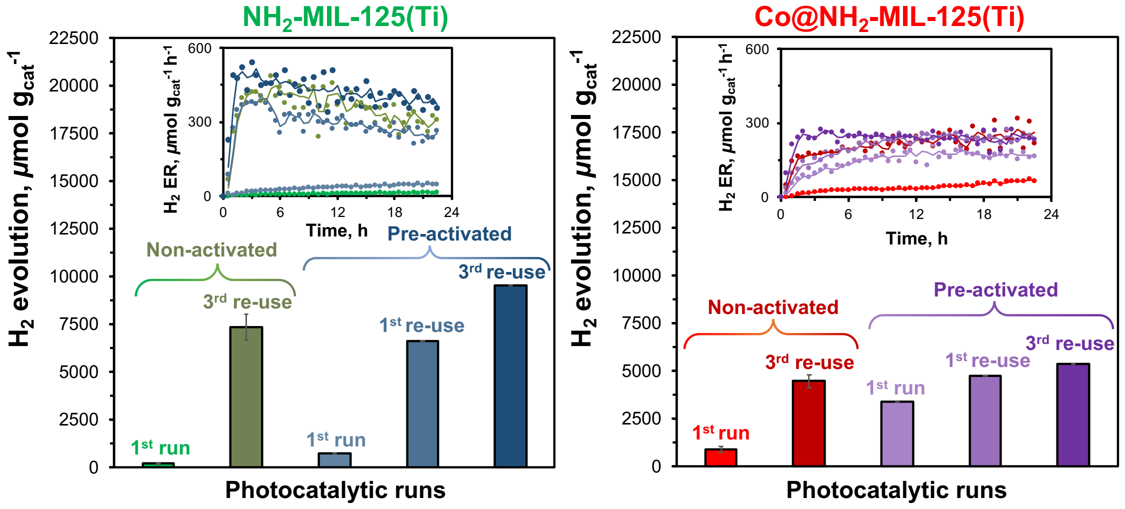

Figure S8. The accumulated amount of evolved hydrogen after 22.5 h of irradiation and H_2 evolution rates (inset) over (Co@)NH_2-MIL-125(Ti), pre-activated and one and three times re-used photocatalysts. Experimental data of H_2 evolution rate are represented by closed circles with a moving average solid line.

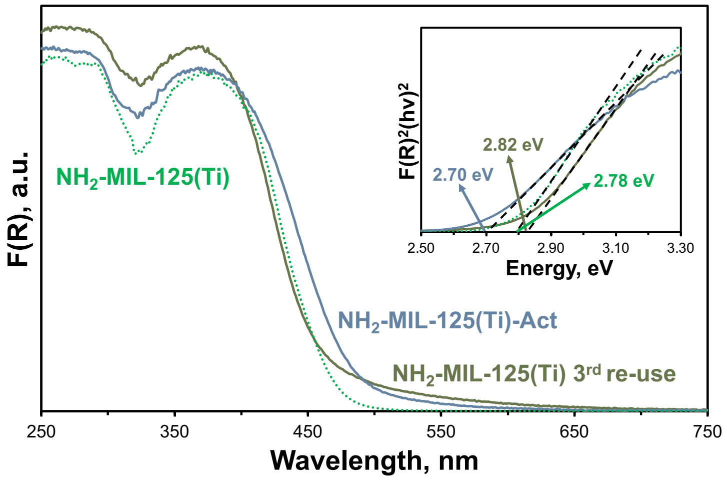

Figure S9. DRUV-Vis spectra of NH_2-MIL-125(Ti) and re-used photocatalysts.

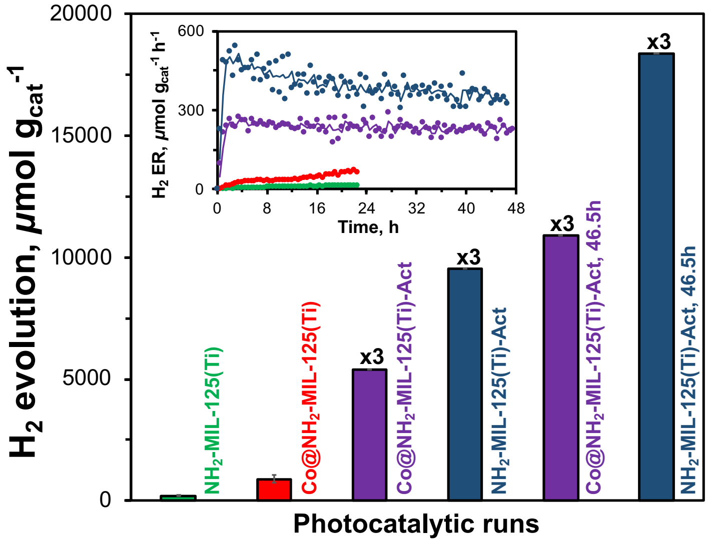

Figure S10. The accumulated amount of evolved H_2 after 22.5 h and 46.5 h of irradiation and H_2 evolution rates (inset) over (Co@)NH_2-MIL-125(Ti) and pre-activated three times re-used (x3) photocatalysts. Experimental data of H_2 evolution rate are represented by closed circles with a moving average solid line.

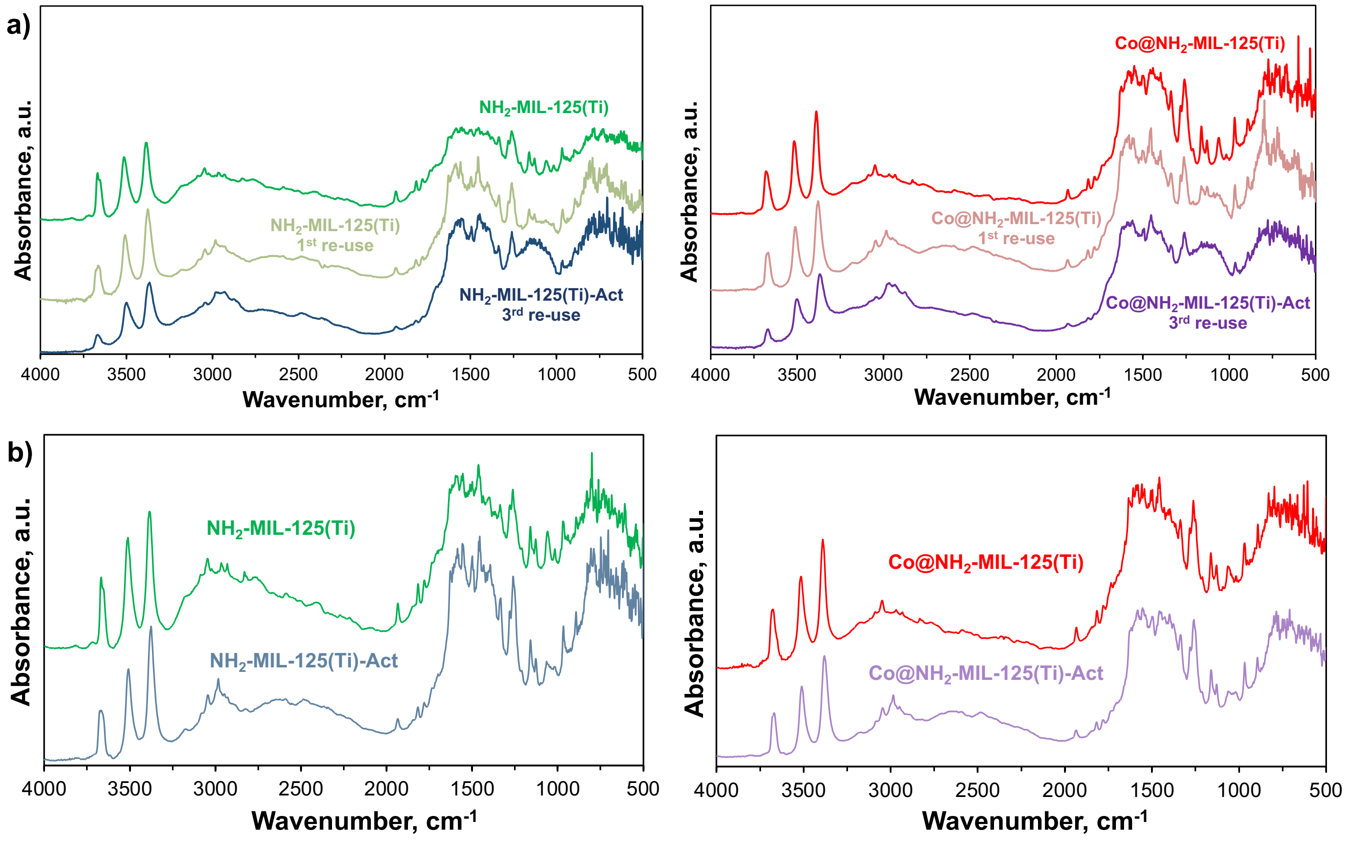

Figure S11. DRIFT spectra of (Co@)NH$_2$-MIL-125(Ti), (a) re-used and (b) pre-activated photocatalysts.

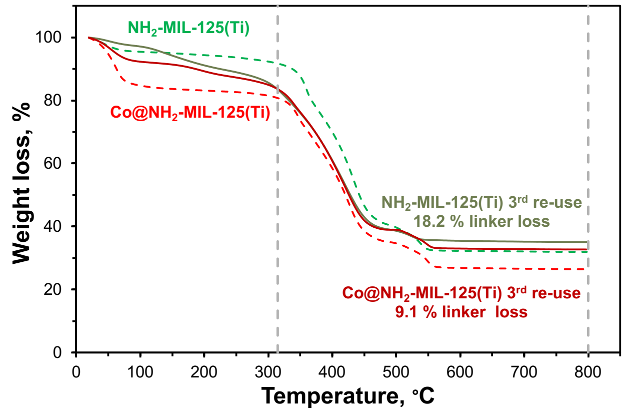

Figure S12. TGA profiles of (Co@)NH$_2$-MIL-125(Ti) (dashed lines) and re-used (solid lines) photocatalysts. The linker loss was calculated within the range of 315-800 ºC.

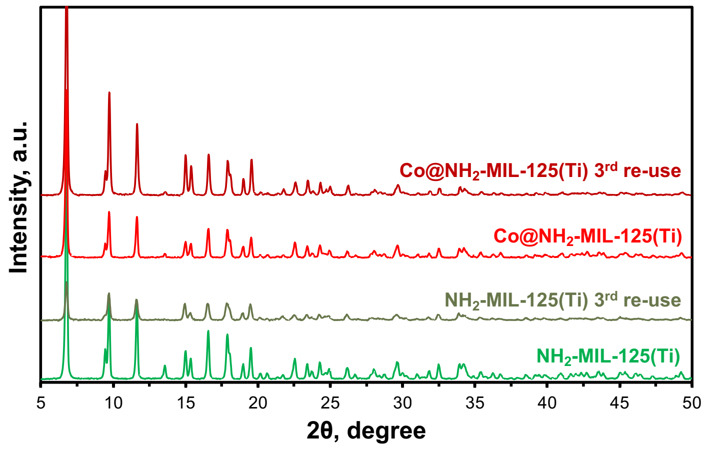

Figure S13. PXRD patterns of pristine and re-used photocatalysts.

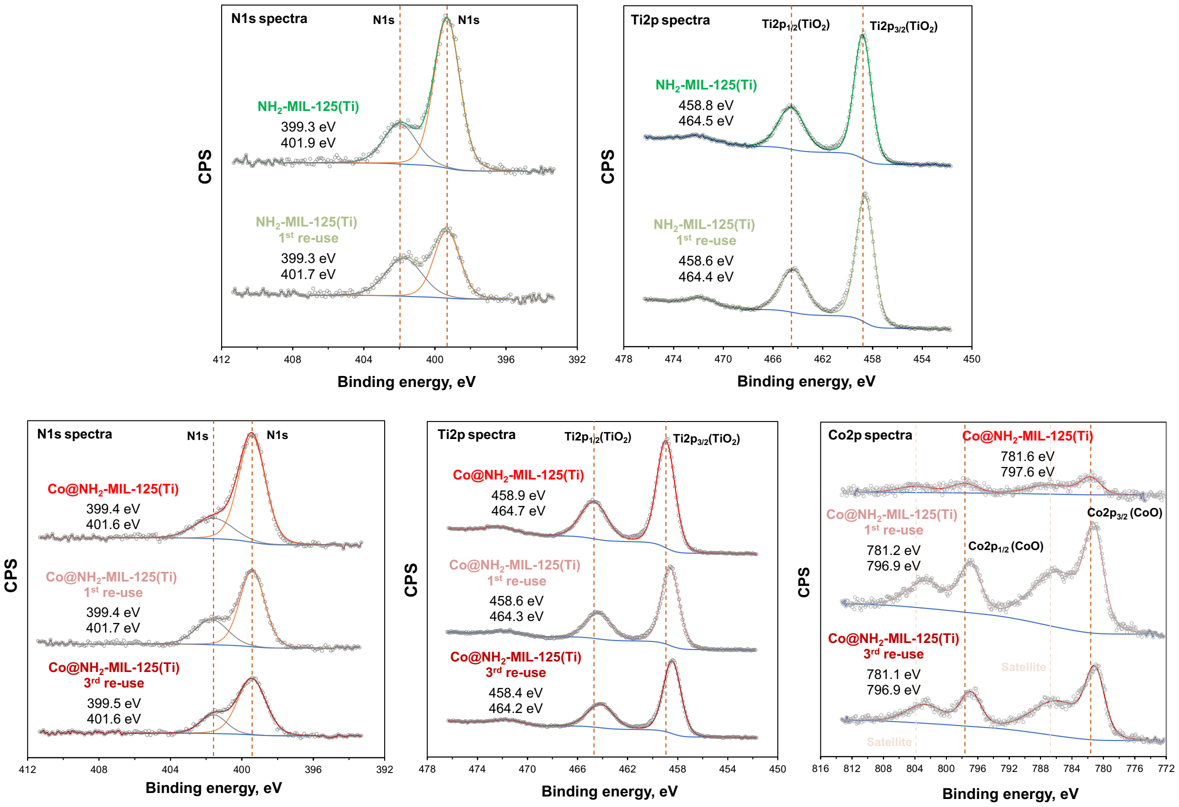

Figure S14. High resolution N1s, Ti2p and Co2p spectra of (Co@)NH_2-MIL-125(Ti) and re-used photocatalysts.

Although no significant alterations of the oxidation state of the constituent elements (Co, Ti, N) were observed on the XPS spectra (Fig. S13), the major differences are found in the element composition of the catalysts, reflected in normalized element ratios (Table S2). A noticeable decrease in N/Ti ratio is observed in spent NH_2-MIL-125(Ti), along with the decrease in number of terminal amino groups, confirming partial linker elimination, particularly from the facial layer of the MOF crystals. Upon introduction of catalytic Co centers in the catalyst, only slightly decrease in N/Ti ratio in reused Co@NH_2-MIL-125(Ti) is noted, while the concentration of Co species on the surface is significantly increased based on N/Co and Ti/Co ratios (Table S2). The latter may imply the migration of Co to the surface of the crystal and/or partial removal of organic linker in conjunction with Ti-oxo cluster from the MOF where Co species are not presented as only negligible changes in cobalt concentration from ICP-OES (Table S3) results were observed.

Table S2. Calculated element ratio of (Co@)NH_2-MIL-125(Ti) based photocatalysts from XPS spectra.

Sample	Element ratio		
	N/Ti^a	N/Co^b	Ti/Co^c
NH_2-MIL-125(Ti)	0.84 ± 0.03	n/a	n/a
NH_2-MIL-125(Ti) 1st re-use	0.48 ± 0.01	n/a	n/a
Co@NH_2-MIL-125(Ti)	0.69 ± 0.01	14.2 ± 0.3	20.5 ± 0.4
Co@NH_2-MIL-125(Ti) 1st re-use	0.72 ± 0.01	2.2 ± 0.1	3.0 ± 0.1
Co@NH_2-MIL-125(Ti) 3rd re-use	0.62 ± 0.02	2.5 ± 0.1	4.1 ± 0.1

^{a} Expected value of N/Ti ratio based on the structural formula $Ti_8O_8(OH)_4(C_6H_3C_2O_4NH_2)_6$ is 0.75;

^{b} Expected value of N/Co ratio based on the structural formula and 1.5 wt% is 14.25;

^{c} Expected value of Ti/Co ratio based on the structural formula and 1.5 wt% is 19.0.

Table S3. ICP-OES results of digested Co@NH_2-MIL-125(Ti) and re-used Co-doped photocatalysts.

Sample	Co content, wt%
Co@NH_2-MIL-125(Ti)	1.53 ± 0.01
Co@NH_2-MIL-125(Ti) 1st re-use	1.27 ± 0.01
Co@NH_2-MIL-125(Ti) 3rd re-use	1.35 ± 0.01

References

(1) Solís, R. R.; Gómez-Avilés, A.; Belver, C.; Rodriguez, J. J.; Bedia, J. Microwave-Assisted Synthesis of NH2-MIL-125(Ti) for the Solar Photocatalytic Degradation of Aqueous Emerging Pollutants in Batch and Continuous Tests. *J Environ Chem Eng* **2021**, *9* (5), 106230. https://doi.org/10.1016/j.jece.2021.106230.

(2) Titantah, J. T.; Lamoen, D. Carbon and Nitrogen 1s Energy Levels in Amorphous Carbon Nitride Systems: XPS Interpretation Using First-Principles. *Diam Relat Mater* **2007**, *16* (3), 581–588. https://doi.org/10.1016/j.diamond.2006.11.048.

(3) Dietrich, P. M.; Graf, N.; Gross, T.; Lippitz, A.; Krakert, S.; Schüpbach, B.; Terfort, A.; Unger, W. E. S. Amine Species on Self-Assembled Monolayers of ω-Aminothiolates on Gold as Identified by XPS and NEXAFS Spectroscopy. In *Surface and Interface Analysis*; John Wiley & Sons, Ltd, 2010; Vol. 42, pp 1184–1187. https://doi.org/10.1002/sia.3224.

(4) Sun, D.; Liu, W.; Fu, Y.; Fang, Z.; Sun, F.; Fu, X.; Zhang, Y.; Li, Z. Noble Metals Can Have Different Effects on Photocatalysis over Metal-Organic Frameworks (MOFs): A Case Study on M/NH2-MIL-125(Ti) (M=Pt and Au). *Chemistry - A European Journal* **2014**, *20* (16), 4780–4788. https://doi.org/10.1002/chem.201304067.

(5) Weidler, N.; Paulus, S.; Schuch, J.; Klett, J.; Hoch, S.; Stenner, P.; Maljusch, A.; Brötz, J.; Wittich, C.; Kaiser, B.; Jaegermann, W. CoOx Thin Film Deposited by CVD as Efficient Water Oxidation Catalyst: Change of Oxidation State in XPS and Its Correlation to Electrochemical Activity. *Physical Chemistry Chemical Physics* **2016**, *18* (16), 10708–10718. https://doi.org/10.1039/c5cp05691h.
